# *CRELD1*-Associated Neurodevelopmental Disorder: Three New Individuals from Unrelated Families

**DOI:** 10.3390/genes16080972

**Published:** 2025-08-18

**Authors:** Jessica Archer, Shuxiang Goh, Christina Miteff, Sheridan O’Donnell, Kristen Park, Himanshu Goel

**Affiliations:** 1 Hunter Genetics, Waratah, NSW 2298, Australia; 2 School of Women and Children’s Health, University of New South Wales, Sydney, NSW 2052, Australia; 3 Department of Neurology, John Hunter Children’s Hospital, New Lambton Heights, NSW 2305, Australia; christina.miteff@health.nsw.gov.au; 4 Departments of Neurology and Paediatrics, Children’s Hospital Colorado, Aurora, CO 80045, USA; 5 School of Medicine and Public Health, University of Newcastle, Callaghan, NSW 2308, Australia

**Keywords:** *CRELD1*, global developmental delay, epileptic encephalopathy, hypotonia, microcephaly

## Abstract

**Background:** *CRELD1* encodes a cell adhesion molecule initially implicated in atrioventricular septal defects (AVSDs). More recently, biallelic *CRELD1* variants have been associated with syndromic and non-syndromic neurodevelopmental disorders (NDDs). **Methods:** We describe three individuals from unrelated families with compound heterozygous *CRELD1* variants, identified through exome sequencing. Clinical and genetic data were reviewed to delineate shared and divergent features. **Results:** All three patients presented with developmental delay, intellectual disability, seizures, hypotonia, and dysmorphic facial features. Patient 1 and patient 2 carried a recurrent variant combination previously reported in five individuals, while Patient 3 harboured the recurrent frameshift p.(Gln320Argfs*25) variant in trans with a novel missense variant. The milder clinical course of patient 3 highlights phenotypic heterogeneity. Notably, none of the patients had cardiac anomalies or immunological abnormalities, further expanding the clinical spectrum associated with *CRELD1*. **Conclusion:** Our findings reinforce genotype–phenotype correlations and provide additional evidence that biallelic *CRELD1* variants underlie a distinct autosomal recessive neurodevelopmental disorder, broadening both the phenotypic and genetic spectrum of this emerging syndrome.

## 1. Introduction

*CRELD1* (cysteine-rich with EGF-like domains 1) encodes a highly conserved transmembrane protein characterised by epidermal growth factor (EGF)-like domains, calcium-binding motifs, and a tryptophan and glutamate rich (WE) domain. It is most abundantly expressed in the developing foetal brain, heart, branchial arches, and limb buds, with continued expression in the adult brain, heart, and skeletal muscle, suggesting pleiotropic roles in organogenesis and tissue homeostasis [1].

The gene was originally identified through positional cloning in infants with chromosome 3p deletions and congenital atrioventricular septal defects (AVSDs) [2]. Heterozygous missense variants in *CRELD1* have been shown to account for approximately 5% of simplex AVSD cases, with incomplete penetrance. The encoded protein functions, in part, through VEGFA-mediated activation of the calcineurin/NFATc1 pathway, a critical regulator of endocardial cushion remodelling during cardiac development [3,4,5].

More recently, biallelic *CRELD1* variants have been implicated as the cause of a syndromic autosomal recessive neurodevelopmental disorder featuring early-onset epilepsy, hypotonia, global developmental delay, and variably associated craniofacial and cardiac features. Functional studies in *Drosophila* and mammalian models have supported a mechanistic link to disrupted ER–mitochondrial tethering, mitochondrial fragmentation, and altered calcium signalling [1,6].

Here, we report three additional individuals from unrelated families with compound heterozygous *CRELD1* variants. These cases further expand the clinical and genetic spectrum of *CRELD1*-related disease and reinforce its relevance in syndromic epileptic encephalopathies.

## 2. Materials and Methods

Three individuals from unrelated families with compound heterozygous *CRELD1* variants were collected via GeneMatcher. Clinical data including detailed phenotypic assessment, neuroimaging results, and genetic information were collated for these individuals. All individuals underwent trio whole exome sequencing using massively parallel sequencing using blood derived DNA. Library preparation was performed using a TWIST BioScience Library Preparation EF KIT (TWIST-Alliance) kit, with libraries analysed on an Illumina NovaSeq 6000. Reads are aligned to Human Genome Reference Sequence GRCh38 or GRCh37 and single nucleotide and short insertion/deletion variants identified using the Dragen Server in Illumina Basespace DRAGEN Enrichment 3.9.5. Variant filtering, prioritisation and reporting were performed using the Genomics Annotation and Interpretation Application (GAIA) in-house pipeline. Variants were filtered based on inheritance pattern, impact, frequency, zygosity and in silico pathogenicity scores. The data analysis pipeline was based on Gemini v18 with annotation from VEP and dbNSFP. Copy Number Variants (CNV) were identified by three CNV callers: CoNIFER, DECoN and XHMM. Mosaicism below ~10% VAF could not be excluded. Variants were classified according to American College of Medical Genetics (ACMG) criteria [7].

## 3. Results

### 3.1. Patient 1

The proband was the first child of unaffected, non-consanguineous parents. He was born at term via forceps-assisted vaginal delivery following an uneventful pregnancy. At birth, weight and length were appropriate for gestational age (25th and 50th percentiles, respectively), while head circumference was on the 3rd percentile. He required continuous positive airway pressure (CPAP) respiratory support for 4 h and was treated with intravenous antibiotics for risk factors for neonatal sepsis. He was circumcised by parental choice. Feeding difficulties were evident from birth, and a videofluoroscopic swallow study (VFSS) at four months identified severe oropharyngeal dysphagia with silent aspiration. He is currently dependent on nasogastric tube feeding, with percutaneous endoscopic gastrostomy placement planned.

At 6.5 months of age, his growth parameters were tracking similarly, with head circumference remaining in the 3rd percentile, height in the 50th percentile, and weight between the 10th and the 25th percentile. His neurodevelopmental phenotype included hypotonia and global developmental delay. At 8 months of age, he was only able to hold his head up for 3 s, rolling one way, cooing and not yet tracking across his field of vision.

**Figure 1:**
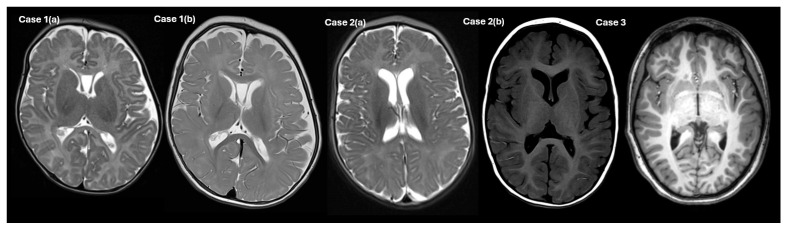
MRI brain images. Comparison of axial images of Case 1 at 3 months (a, T2 axial) and 7 months (b, T2 axial), demonstrating delayed white matter myelination, white matter volume loss and prominent cavum septum pellucidum. Case 2 at 5 months (a, T2 axial) and 20 months (b, T1 axial), showing progression of white matter loss. Case **3’s** MRI brain was normal.

He was diagnosed with myoclonic epilepsy at six months of age, manifesting as bilateral lower limb jerking. EEG demonstrated central midline epileptiform discharges correlating with myoclonic events. He has required two hospital admissions for seizure management, including one ICU admission, and is currently on dual antiepileptic therapy. Additional features include unilateral strabismus, for which he is undergoing occlusion therapy. Brain MRIs at 3 and 7 months of age demonstrated delayed white matter myelination and prominent cavum septum pellucidum (Figure 1).

**Figure 2:**
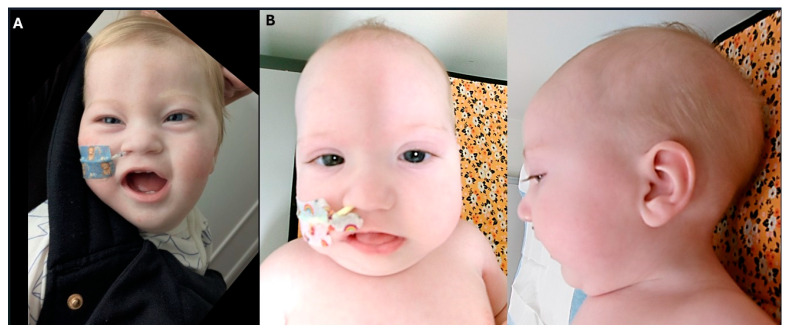
Photographs of two individuals with *CRELD1*-related disorder: (**A**) Patient 1 at 10 months of age; (**B**) front and profile photos of Patient 2 at 8 months of age.

Cardiological assessment is pending; however, no abnormalities were detected during an extended period of rhythm monitoring in ICU. Dysmorphic features included a thin upper lip, smooth and thin philtrum, long upslanting palpebral fissures, bitemporal narrowing, high forehead and prominent metopic ridge (Figure 2A).

SNP microarray and urine metabolic screen were non-diagnostic. Trio whole exome sequencing (ES) identified compound heterozygous variants in *CRELD1*. GRCH38 assembly and NM_015513.4 transcript were used. A maternally inherited missense variant c.575G > A; p.(Cys192Tyr) and a paternally inherited frameshift variant c.959del; p.(Gln320Argfs*25) were identified. Both variants were previously described in the literature [6]. ACMG criteria used for c.575G > A; p.(Cys192Tyr) were PM3_moderate, PS4_moderate, PM2_supporting, PP3_supporting. ACMG criteria used for c.959del; p.(Gln320Argfs *25) were PVS1_strong, PS4_moderate and PM2_supporting.

### 3.2. Patient 2

The proband is the third child of unaffected parents, with two healthy siblings. She was born at term by planned caesarean section due to prior caesarean sections, following an uncomplicated pregnancy aside from maternal aspirin use for preeclampsia prophylaxis. Initial postnatal adaptation included transient CPAP support. Birth parameters were between the 40th and 60th percentiles. A cleft palate involving both soft and hard components was diagnosed postnatally. Audiometry demonstrated resolving conductive hearing loss. Due to poor feeding and vomiting, she required nasogastric and later nasojejunal tube feeding; a percutaneous endoscopic jejunostomy is planned. Despite early growth faltering, she has tracked along the 25th percentile for weight and 50th percentile for height. Head circumference declined postnatally, falling below the 3rd percentile by one year.

Seizure onset occurred at 3.5 months in the context of febrile illness. EEG revealed 3 Hz spike-and-wave discharges with eyelid myoclonia. Despite trials of multiple antiepileptics and cannaboid oil, she continues to experience > 15 seizures/day by 7 months of age.

Her neurodevelopmental profile includes severe hypotonia and global developmental delay. At 8 months, she had limited head control, could roll prone to supine, grasp objects, and vocalise. She required full postural support for sitting or standing.

Cardiac evaluation revealed a small patent ductus arteriosus on early echocardiography, with normal repeat imaging and Holter monitoring at 14 months. Immunological workup was normal, including appropriate T and B cell function and response to vaccines. She has had several self-limited viral illnesses but no evidence of immune deficiency. Brain MRIs at 5 months and 20 months of age demonstrated some white matter loss.

Dysmorphic features include a single palmar crease, mild pectus excavatum, retrognathia, glossoptosis, and facial characteristics such as high forehead, flat midface, broad nasal bridge, epicanthal folds, downturned corners of mouth, sparse arched eyebrows, and tented thin upper lip, suggestive of possible myopathic facies.

SNP microarray and metabolic screen were unremarkable. Trio ES demonstrated the same two variants as in patient #1, a maternally inherited missense variant, NM_015513.4: c.575G > A; p.(Cys192Tyr) and paternally inherited frameshift variant c.959del; p.(Gln320Argfs*25).

### 3.3. Patient 3

The proband was born at term following an uncomplicated pregnancy. Birth weight was on the 50th percentile. There were no neonatal complications other than jaundice requiring phototherapy. Speech delays were noted at 2 years of age with few words and limited 2-word combinations. No concerns were raised regarding gross or fine motor development. She has not undergone formal cognitive assessment; however, is estimated to function at the level of an elementary schooler in adolescence. She has mild hypotonia and reports sleeping and behavioural anomalies. She has thin fingers with decreased intrinsic hand muscle definition. There are no concerns regarding her vision or hearing.

She had her first seizure at 4 years of age in the context of a febrile illness. Her first unprovoked seizure was at the age of 10 years. Her predominant seizure type is generalised tonic–clonic and EEG revealed generalised spikes and polyspikes in runs at 2–3 Hz with photoparoxysmal response and mild background slowing. At age 16 years, her seizures are described as intractable. Lamotrigine was possibly associated with worsening of her seizure control. She has had normal MRI brain of her brain on two occasions.

She has no feeding or growth issues. At 15 years 9 months, her weight and head circumference were approximately 75th percentile, and height was 155 cm (13th percentile). She has achieved puberty with menarche aged 12 years. She has had a normal ECG and echocardiogram. Results of Holter monitoring are not available.

Whole exome sequencing identified compound heterozygous variants in *CRELD1*, comprising a paternally inherited frameshift variant NM_015513.4: c.959del; p.(Gln320Argfs*25), and a maternally inherited missense variant c.800G > C; p.(Gly267Ala) that has not previously been reported. The p.(Gly267Ala) is absent from gnomAD database and lies within a protein region that is highly intolerant to missense variants. It lies within the first calcium binding EGF-like protein domain and is proximal to previously reported pathogenic missense variant p.(Cys262Arg), suggesting functional relevance of this region [6]. ACMG criteria used were: PM2_supporting, PP3_strong and PM3_moderate.

## 4. Discussion

Biallelic disease causing variants (DCVs) in *CRELD1* have recently been recognised as the cause of a neurodevelopmental disorder that typically presents with early-onset epilepsy, hypotonia, feeding difficulties, and developmental delay, often accompanied by craniofacial and cardiac anomalies [6]. These findings underscore the gene’s critical role in both cardiac and neural development, primarily through its involvement in the VEGFA-dependent calcineurin/NFATc1 signalling pathway, a key regulator of endocardial cushion remodelling and cardiac morphogenesis [3,4,8]. The expanding spectrum of phenotypes associated with *CRELD1* variants highlights the pleiotropic nature of the gene, influencing multiple organ systems, including the brain, heart, and craniofacial structures.

The CRELD1 protein, particularly isoform 2 (NP_056328.3), is a highly conserved transmembrane protein with essential EGF-like and calcium-binding domains. These domains are crucial for protein stability, cellular function, and inter-organelle communication, particularly between the endoplasmic reticulum (ER) and mitochondria. Studies in model organisms have demonstrated that loss-of-function variants in Creld1 disrupt ER-mitochondrial tethering, leading to mitochondrial fragmentation, impaired calcium signalling, and oxidative stress. These cellular defects are hypothesised to contribute to the neuromuscular and epileptic phenotypes observed in patients with *CRELD1*-related disease. The marked hypotonia, early-onset seizures, and white matter abnormalities observed in our Patient 1 support this model and suggest that mitochondrial dysfunction may play a central role in disease pathogenesis [1].

The clinical findings in our three patients are consistent with the previously described neurodevelopmental disorder associated with biallelic *CRELD1* variants. Patients 1 and 2 exhibit profound hypotonia, developmental delay, feeding difficulties, early-onset epilepsy, and microcephaly, along with dysmorphic craniofacial features, including cleft palate and flat midface, a manifestation that further supports the role of *CRELD1* in craniofacial development. These observations are in line with previous reports of *CRELD1*-related syndromes, reinforcing the gene’s involvement in both brain and craniofacial tissue development [6]. Patient 3, notably, presents a milder phenotype with developmental delay, intractable seizures, and mild hypotonia, highlighting the phenotypic variability and suggesting potential residual protein function from the novel missense allele.

The two recurrent variants identified in patients 1 and 2, c.575G > A, p.(Cys192Tyr) and c.957del, p.(Gln320Argfs25), were previously reported in five other individuals from four unrelated families, showing a consistent phenotype across diverse genetic backgrounds. The p.(Cys192Tyr) substitution lies within an EGF-like domain, where it likely disrupts the critical disulfide bond formation, compromising protein stability and function. The p.(Gln320Argfs25) frameshift variant is predicted to result in a truncated protein, either leading to nonsense-mediated decay or loss of key functional domains. These variants support a strong genotype–phenotype correlation and affirm the pathogenicity of this allele combination in the context of *CRELD1*-related neurodevelopmental disorders [6]. The recurrence of the same variant pair in now six unrelated families raises the possibility of a founder effect or mutational hotspot and the rarity of other pathogenic frameshift or missense variants in *CRELD1*. p.(Cys192Tyr) and p.(Gln320Argfs25) are also the two *CRELD1* variants with the highest allele counts in gnomAD and were found predominantly in non-Finnish European populations^6^, further supporting a founder effect.Comparatively, individuals with the p.(Gln320Argfs25) variant in trans with other missense alleles (e.g., p.(Asp386Asn), p.(Cys262Arg), and the novel p.(Gly267Ala) in Patient 3) have not consistently exhibited microcephaly or feeding difficulties, though seizures were universally present. This variation underscores allelic heterogeneity within *CRELD1*-related disorders [6].

Cardiac monitoring remains a key aspect of clinical management. Although congenital heart defects are variably reported in patients with biallelic *CRELD1* variants, conduction abnormalities such as bradycardia and prolonged PR intervals have been observed more frequently than structural anomalies [6]. While none of our patients exhibited cardiac involvement initially, ongoing surveillance was implemented, particularly given the known role of *CRELD1* in heart development and conduction pathways.

Immunological involvement, while biologically plausible given *CRELD1*’s influence on calcineurin/NFAT signalling, a pathway integral to T cell development, has not been consistently documented. Recurrent infections have been previously observed; however, none of our patients experienced more illnesses than expected for their age. Immunological testing of Patient 2 was performed solely due to previous reports of immunological involvement in *CRELD1*-related disorder and was within normal parameters, which mirrors prior observations [6].

Finally, the identification of *CRELD1* variants in all three of our cases was enabled by exome sequencing. This highlights the limitations of phenotype-first diagnostic approaches and emphasises the value of unbiased genomic testing in rare, syndromic neurodevelopmental disorders.

## 5. Conclusions

We report three additional individuals with biallelic *CRELD1* variants, including a novel missense variant and several previously under-recognised clinical features. Our findings contribute to the expanding understanding of *CRELD1*-associated neurodevelopmental disorder, reinforce genotype–phenotype correlations, and support a broader, more nuanced view of the clinical manifestations of this rare disorder.

## Data Availability

Data supporting reported results available on request from corresponding author.

## Abbreviations

The following abbreviations are used in this manuscript:

AVSD	Atrioventricular Septal Defects
NDD	Non-syndromic Neurodevelopmental Disorders
EGF	Epidermal Growth Factor
CPAP	Continuous Positive Airway Pressure
VFSS	Videofluoroscopic Swallow Study
EEG	Electroencephalogram
ICU	Intensive Care Unit
MRI	Magnetic Resonance Imaging
SNP	Single Nucleotide Polymorphism
ACMG	American College of Medical Genetics and Genomics
ES	Exome Sequencing
ECG	Electrocardiogram
DCV	Disease-Causing Variants
ER	Endoplasmic Reticulum
gnomAD	Genome Aggregation Database
